# Effect of catheter-based renal denervation on left ventricular function, mass and (un)twist with two-dimensional speckle tracking echocardiography

**DOI:** 10.1007/s12574-017-0336-6

**Published:** 2017-05-11

**Authors:** Lida Feyz, Bas M. van Dalen, Marcel L. Geleijnse, Nicolas M. Van Mieghem, Ron T. van Domburg, Joost Daemen

**Affiliations:** Department of Cardiology, Thoraxcenter, Erasmus University Medical Center, P.O. Box 2040, 3000 CA Rotterdam, The Netherlands

**Keywords:** Speckle tracking echocardiography, Twist, Strain, Renal sympathetic denervation

## Abstract

**Background:**

Speckle tracking echocardiography (STE) is an echocardiography modality that is able to measure left ventricular (LV) characteristics, including rotation, strain and strain rate. Strain measures myocardial fibre contraction and relaxation. This study aims to assess the effect of renal sympathetic denervation (RDN) on functional myocardial parameters, including STE, and to identify potential differences between responders and non-responders.

**Methods:**

The study population consisted of 31 consecutive patients undergoing RDN in the context of treatment for resistant hypertension. Patients were included between December 2012 and June 2014. Transthoracic echocardiography and speckle tracking analysis was performed at baseline and at 6 months follow-up.

**Results:**

The study population consisted of 31 patients with treatment-resistant hypertension treated with RDN (mean age 64 ± 10 years, 15 men). The total study population could be divided into responders (*n* = 19) and non-responders (*n* = 12) following RDN. RDN reduced office blood pressure by 18.9 ± 26.8/8.5 ± 13.5 mmHg (*p* < 0.001). A significant decrease was seen in LV posterior wall thickness (LVPWd) (0.47 ± 1.0 mm; *p* = 0.020), without a significant change in the LV mass index (LVMI). In the total cohort, only peak late diastolic filling velocity (A-wave velocity) decreased significantly by 5.3 ± 13.2 cm/s (*p* = 0.044) and peak untwisting velocity decreased significantly by 14.5 ± 28.9°/s (*p* = 0.025).

**Conclusion:**

RDN reduced blood pressure and significantly improved functional myocardial parameters such as A-wave velocity and peak untwisting velocity in patients with treatment-resistant hypertension, suggesting a potential beneficial effect of RDN on myocardial mechanics.

## Background

Hypertension is associated with a significantly increased risk for adverse cardiac and cerebrovascular events, as well as chronic kidney disease [1]. Despite optimal medical treatment, blood pressure control often remains poor and the risk for cardiovascular disease remains high. With prevalence ranging between 15 and 30%, treatment-resistant hypertension remains an important medical challenge and leads to intrinsic changes in the heart muscle and is associated with left ventricular (LV) hypertrophy and diastolic dysfunction [2, 3].

Catheter-based renal denervation (RDN) has been introduced as a treatment modality to optimise blood pressure control in patients with treatment-resistant hypertension by reducing sympathetic nerve activity. Unfortunately, the exact blood pressure-lowering effect of renal sympathetic denervation remains disputed, demonstrated by non-responder rates varying between 8 and 37%, depending on study-specific cohorts and definitions used [4]. However, sympathetic hyperactivity has been directly associated with LV remodelling and heart rate, which makes it imperative to look at the effects of renal sympathetic denervation beyond blood pressure [5]. Speckle tracking echocardiography (STE) is a new echocardiography modality that is able to measure LV characteristics, including rotation, strain and strain rate. Strain measures myocardial fibre contraction and relaxation [6]. This study aims to assess the effect of RDN on functional myocardial parameters, including STE, and to identify potential differences between responders and non-responders.

## Methods

### Study population

The study population consisted of 31 consecutive patients undergoing RDN in the context of treatment for resistant hypertension according to recent recommendations [7]. Patients were included between December 2012 and June 2014. All patients underwent non-invasive pre-procedural renal artery imaging and were discussed in a multi-disciplinary team including interventional cardiologists, radiologists and hypertension specialists. As part of routine practice, all patients referred for RDN underwent extensive blood and urine analyses, 24-h ambulatory blood pressure measurement (24 h ABPM), echocardiography, echocardiogram (ECG) and magnetic resonance imaging (MRI) to assess renal artery eligibility and exclude renal artery stenosis in order to be able to exclude secondary causes of hypertension. Informed consent was obtained from all patients.

### Definitions and end-points

Office blood pressure measurements were recorded three times in a resting situation with intervals of 5 min using an Omron automated blood pressure monitor. Patients were classified as responders in case the drop in 6 months office systolic blood pressure was 10 mmHg or higher. In order to identify subtle changes in LV function, STE was used to obtain apical rotation, basal rotation, LV twist, twist velocity, peak untwisting velocity, time to peak untwisting velocity, global longitudinal strain (GLS), global circumferential strain (GCS), peak early and late longitudinal diastolic strain rate, and peak early and late circumferential diastolic strain rate. LV twist is defined as the maximal value of the apical systolic rotation–basal systolic rotation [8].

### Transthoracic echocardiography

Echocardiography measurements were performed before the RDN procedure (baseline) and 6 months after the RDN procedure. Two-dimensional grey-scale images were obtained in the left lateral decubitus position using a commercially available ultrasound system (iE33, Philips, Best, The Netherlands), equipped with a broadband (1–5 MHz) S5-1 transducer (frequency transmitted 1.7 MHz, received 3.4 MHz). Data were analysed by two experienced echocardiographers according to the recent recommendations [9]. The following echocardiographic parameters were acquired: LV end-diastolic septal (LVSd) and posterior wall thickness (LVPWd), and LV end-diastolic (LVEDD) and end-systolic dimension (LVESD). LV mass was calculated with the Devereux formula [10]. Body surface area (BSA) was calculated according to the Mosteller formula [11]. LV mass was indexed by BSA as recommended in the guidelines [12].

From the mitral inflow pattern, peak early (E-wave velocity) and late (A-wave velocity) filling velocities, E/A ratio and E-wave velocity deceleration time were measured. Tissue Doppler was applied end-expiratory in the pulsed-wave Doppler mode at the level of the inferoseptal side of the mitral annulus from an apical four-chamber view, to obtain Em septal (peak early diastolic wave velocity of the mitral annulus) and E/Em ratio.

To acquire the highest wall tissue velocities, the angle between the Doppler beam and the longitudinal motion of the investigated structure was adjusted to a minimal level. The spectral pulsed-wave Doppler velocity range was adjusted to obtain appropriate scale.

To optimise STE, the settings were adjusted to obtain a frame rate of 50–70 frames/s. The echo images were transformed to a QLAB Advanced Quantification Software workstation (version 10.0, Philips, Best, The Netherlands) for offline analysis.

### Speckle tracking analysis

STE is an approved echocardiographic modality that provides information on regional and global ventricular function [13]. In order to obtain this information, apical long-axis views (four-, three- and two-chamber views) and parasternal short-axis views (at the apical, mid-ventricular and basal LV levels) were assessed. The aortic valve closure was assessed in a parasternal long-axis view and added manually. After selecting the appropriate view, the endocardial border was automatically recognised and the tracking points were positioned. When this auto-trace function was not optimal, the tracking points were re-positioned manually on an end-diastolic frame. Next, the software automatically tracked these points using speckle tracking. LV ejection fraction was assessed using this automated endocardial border detection. Most components of LV systolic function [rotation (clockwise rotation as viewed from the apical level has a positive value and counterclockwise rotation from the basal level has a negative value), twist, global circumferential strain (GCS) and GCS rate (GCSR)] and diastolic function (peak untwisting velocity, time to peak untwisting velocity, peak early and late circumferential diastolic strain rate) were abstracted from parasternal short-axis views, whereas others were derived from the apical views [systolic function: global longitudinal strain (GLS) and GLS rate (GLSR); diastolic function: peak early and late longitudinal diastolic strain rate]. Data were exported to a spreadsheet program (Excel, Microsoft Corporation, Redmond, WA) to determine these parameters. In a previous study, we have demonstrated the reproducibility and variability of the parameters investigated in the current study in our centre [14].

### RDN procedure

Procedures were performed using four different systems: Paradise™ (ReCor Medical, Palo Alto, CA) (*n* = 13), OneShot™ (Covidien, Campbell, CA) (*n* = 3), Vessix V2™ (Boston Scientific, Natick, MA) (*n* = 5) and Symplicity™ (Medtronic, Minneapolis, MN) (*n* = 10). Procedures were performed according to the device-specific instructions for use [7, 15]. All procedures were performed under conscious sedation with midazolam and fentanyl.

### Statistical analysis

All continuous variables are expressed as mean ± standard deviation (SD). Continuous variables were compared using Student’s *t* test. Simple linear regression of peak untwisting velocity against heart rate was performed. Categorical variables were compared with the Chi square test or Fisher’s exact test when appropriate. A *p*-value <0.05 was considered statistically significant. The statistical analysis was performed with SPSS software (version 21.0).

## Results

### Study population

Thirty-one patients with resistant hypertension following RDN were enrolled in this study, all of whom completed the 6 months follow-up period. The mean age was 64 ± 10 years and 15 patients (48%) were male. A total of 19 patients were classified as responders versus 12 non-responders. Besides a significantly lower age of the responders (61 ± 10 vs. 69 ± 9 years in the non-responders; *p* = 0.028), no significant differences in patient characteristics were seen between both groups (Table 1).

**Table 1:** Clinical characteristics of the study population, responder vs. non-responder at baseline

	All patients	Responder	Non-responder	*p*-Value
	Baseline (*n* = 31)	Baseline (*n* = 19)	Baseline (*n* = 12)	Responder vs. non-responder
Age (years)	64 ± 10	61 ± 10	69 ± 9	0.028
Male gender, *n* (%)	15 (48)	10 (53)	5 (42)	0.552
BMI (kg/m^2^)	29 ± 4	29 ± 5	28 ± 3	0.639
Mean office SBP (mmHg)	182 ± 18	186 ± 20	176 ± 15	0.133
Mean office DBP (mmHg)	94 ± 16	97 ± 14	89 ± 18	0.149
Mean systolic ABPM (mmHg)	150 ± 12	148 ± 12	152 ± 13	0.358
Mean diastolic ABPM (mmHg)	83 ± 13	83 ± 11	82 ± 17	0.946
Heart rate (beats/min)	68 ± 12	69 ± 12	65 ± 12	0.282
CAD (%)	16 (52)	12 (63)	4 (33)	0.106
Atrial fibrillation (%)	2 (7)	2 (11)	–	0.368
Cardiovascular risk factors, *n* (%)				
Hypercholesterolaemia	22 (71)	15 (79)	7 (58)	0.204
Current smoker	11 (36)	6 (32)	5 (42)	0.619
Diabetes mellitus	8 (26)	7 (37)	1 (8)	0.086
Number of hypertensive drugs	4 ± 1	4 ± 1	4 ± 1	0.634
Patients receiving (drug class) (%)				
ACE inhibitors/ARBs	27 (87)	17 (90)	10 (83)	0.507
Direct renin inhibitors	1 (3)	–	1 (8)	0.387
Beta-blockers	25 (81)	16 (84)	9 (75)	0.435
Alpha-blockers	9 (29)	4 (21)	5 (42)	0.168
Calcium channel blockers	25 (81)	16 (84)	9 (75)	0.435
Aldosterone antagonist	4 (13)	3 (16)	1 (8)	0.493
Diuretics	22 (71)	13 (68)	9 (75)	0.417
Central acting agent	3 (10)	1 (5)	2 (17)	0.328

Values are mean ± standard deviation (SD) or *n* (%)

*BMI* Body mass index; *SBP* systolic blood pressure; *DBP* diastolic blood pressure; *ABPM* ambulatory blood pressure monitoring; *CAD* coronary artery disease; *ACE* angiotensin-converting enzyme; *ARBs* angiotensin receptor blocker

### Blood pressure

A significant decrease was seen in office-based systolic blood pressure (SBP) and diastolic blood pressure (DBP) after RDN at 6 months follow-up: 182 ± 18 vs. 163 ± 27 mmHg (*p* < 0.001) and 94 ± 16 vs. 85 ± 14 mmHg (*p* = 0.001), respectively. The same applied for the systolic 24 h ABPM after RDN (150 ± 12  vs. 142 ± 18 mmHg; *p* = 0.017) and the diastolic 24 h ABPM after RDN (83 ± 13 vs. 78 ± 11 mmHg; *p* = 0.006) (Table 2).

**Table 2:** Clinical, echocardiographic and speckle tracking parameters in patients following renal denervation

	Baseline	Follow-up (6 months)	*p*-Value
Clinical parameters			
Mean office SBP (mmHg)	182 ± 18	163 ± 27	<0.001
Mean office DBP (mmHg)	94 ± 16	85 ± 14	0.001
Mean systolic ABPM (mmHg)	150 ± 12	142 ± 18	0.017
Mean diastolic ABPM (mmHg)	83 ± 13	78 ± 11	0.006
Heart rate (beats/min)	68 ± 12	63 ± 11	0.016
Echocardiographic parameters			
LA size (mm)	45.1 ± 7.9	44.5 ± 6.8	0.523
LAVI (mL/m^2^)	36.8 ± 10.9	35.6 ± 11.0	0.335
IVSd (mm)	10.8 ± 2.1	10.3 ± 1.8	0.105
LVPWd (mm)	8.8 ± 1.4	8.3 ± 1.5	0.020
LVEDD (mm)	53.8 ± 8.1	55.5 ± 7.9	0.028
LVESD (mm)	37.7 ± 8.7	39.5 ± 8.3	0.048
LVEF (%)	59.5 ± 11.0	58.1 ± 10.1	0.123
LVMI (g/m^2^)	105.8 ± 33.8	102.6 ± 30.2	0.133
Doppler indices			
E (cm/s)	67.2 ± 20.5	65.0 ± 23.1	0.519
A (cm/s)	69.7 ± 14.2	64.4 ± 14.0	0.044
E/A ratio	1.0 ± 0.3	1.0 ± 0.4	0.557
DET (ms)	219.7 ± 51.6	219.7 ± 84.3	0.996
Em septal (cm/s)	5.5 ± 1.8	5.1 ± 1.7	0.134
E/Em ratio	12.3 ± 4.7	12.5 ± 3.0	0.840
Speckle tracking echocardiography			
GLS (%)	−19.6 ± 4.2	−19.9 ± 3.5	0.653
GCS (%)	−27.3 ± 6.5	−27.2 ± 5.3	0.877
Early GLSR	0.89 ± 0.25	0.89 ± 0.21	0.932
Late GLSR	0.80 ± 0.23	0.82 ± 0.24	0.495
Early GCSR	1.88 ± 0.62	1.80 ± 0.51	0.470
Late GCSR	1.39 ± 0.50	1.32 ± 0.44	0.432
Apical GR (°)	4.8 ± 3.4	4.3 ± 2.7	0.542
Basal GR (°)	−4.2 ± 2.0	−3.5 ± 2.2	0.212
Twist (°)	8.7 ± 4.4	7.4 ± 3.7	0.178
Twist velocity (°/s)	15.5 ± 12.9	13.7 ± 12.1	0.665
Peak untwisting velocity (°/s)	−70.6 ± 28.5	−56.1 ± 24.9	0.025
Time to peak untwisting velocity (s)	0.08 ± 0.07	0.09 ± 0.08	0.707
LVEDV (mL)	76.1 ± 28.4	82.6 ± 34.2	0.152
LVESV (mL)	30.5 ± 17.9	33.9 ± 22.9	0.146
LVEF (%)	61.8 ± 10.1	61.5 ± 8.5	0.803

Values are mean ± SD

*SBP* Systolic blood pressure; *DBP* diastolic blood pressure; *ABPM* ambulatory blood pressure monitoring; *LA* left atrial; *LAVI* left atrial volume indexed; *IVSd* interventricular septum thickness (diastole); *LVPWd* left ventricular posterior wall thickness (diastole); *LVEDD* left ventricular end-diastolic dimension; *LVESD* left ventricular end-systolic dimension; *LVEF* left ventricular ejection fraction; *LVMI* left ventricular mass index; *E* peak early phase filling velocity; *A* peak atrial phase filling velocity; *DET* deceleration time; *Em* peak early wave velocity; *GLS* global longitudinal strain; *GCS* global circumferential strain; *GLSR* global longitudinal strain rate (early and late diastole); *GCSR* global circumferential strain rate (early and late diastole); *GR* global rotation (apical and basal level). Twist is defined as the instantaneous left ventricular peak systolic twist. The peak untwisting velocity is the peak diastolic de-rotation velocity. *EDV* End-diastolic volume; *ESV* end-systolic volume

### Conventional echocardiography and STE

In the total cohort, significant differences at baseline versus the 6-month follow-up period were noted in LVPWd, A-wave velocity and peak untwisting velocity. LVPWd decreased significantly from 8.8 ± 1.4 mm at baseline to 8.3 ± 1.5 mm at follow-up (*p* = 0.020). LV mass index (MI) reduced by 3.2 ± 11.6 g/m^2^ at 6 months. A-wave velocity decreased significantly from 69.7 ± 14.2 cm/s at baseline to 64.4 ± 14.0 cm/s at follow-up (*p* = 0.044). No significant changes were seen in the other conventional echocardiographic parameters. Furthermore, peak untwisting velocity decreased significantly from −70 ± 28.5°/cm at baseline to −56 ± 24.9°/cm at follow-up (*p* = 0.025) (Table 2).

**Fig. 1:**
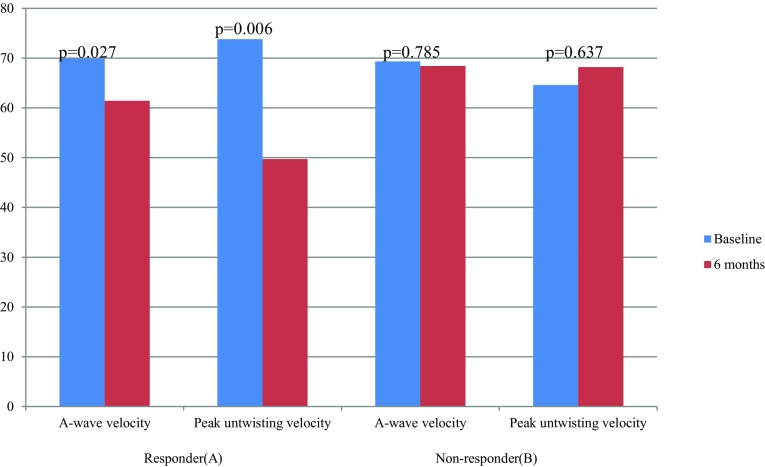
Change in A-wave velocity and peak untwisting velocity at baseline and 6 months follow-up, categorised by responders and non-responders

Stratifying the cohort in responders and non-responders did reveal significant changes from baseline to follow-up in A-wave velocity and peak untwisting velocity in the responders (Fig. 1). In the non-responders, LVESD increased significantly from 37.4 ± 9.3 to 39.7 ± 10.1 mm (*p* = 0.013), although it should be noted that LV ejection fraction (EF) showed no difference. Also, no differences were found in the more sensitive systolic parameters GLS, GCS and LV twist.

Comparing the baseline characteristics of both cohorts, no difference was observed in the LVMI at baseline in the responders as compared to the non-responders (98 ± 25.4 vs. 116 ± 42.9 g/m^2^) (Table 3).

**Table 3:** Baseline and 6-month follow-up parameters in patients following renal denervation, responders vs. non-responders

	Responder (*n* = 19)			Non-responder (*n* = 12)			*p* value
	Baseline	6 months	*p* value	Baseline	6 months	*p* value	Responder vs. non-responder at baseline
Echocardiographic parameters							
LA size (mm)	44.6 ± 8.0	44.2 ± 7.5	0.712	45.8 ± 7.9	44.9 ± 5.7	0.585	0.706
LAVI (mL/m^2)^	37.3 ± 11.1	36.0 ± 12.2	0.394	36.1 ± 11.3	34.9 ± 9.7	0.637	0.855
IVSd (mm)	10.5 ± 2.0	10.3 ± 2.2	0.408	11.3 ± 2.2	10.3 ± 1.2	0.176	0.352
LVPWd (mm)	8.7 ± 1.6	8.3 ± 1.8	0.130	9.0 ± 1.2	8.4 ± 1.2	0.089	0.546
LVEDD (mm)	52.9 ± 7.8	55.1 ± 6.8	0.060	55.1 ± 8.7	56.2 ± 9.7	0.289	0.484
LVESD (mm)	37.9 ± 8.5	39.4 ± 7.3	0.284	37.4 ± 9.3	39.7 ± 10.1	0.013	0.872
LVEF (%)	57.6 ± 10.1	57.0 ± 9.5	0.587	62.5 ± 12.2	59.8 ± 11.1	0.088	0.235
LVMI (g/m^2^)	98.8 ± 25.4	98.8 ± 25.4	^a^	116 ± 42.9	108.5 ± 36.9	0.136	0.151
Doppler indices							
E (cm/s)	72.2 ± 19.5	70.9 ± 24.7	0.759	59.7 ± 20.5	56.2 ± 17.7	0.555	0.103
A (cm/s)	69.9 ± 17.0	61.4 ± 14.4	0.027	69.3 ± 10.0	68.4 ± 13.0	0.785	0.914
E/A ratio	1.0 ± 0.3	1.1 ± 0.3	0.374	0.9 ± 0.3	0.9 ± 0.4	0.889	0.249
DET (ms)	207.5 ± 40.6	189.7 ± 41.7	0.155	238.1 ± 62.2	264.6 ± 111.3	0.400	0.113
Em septal (cm/s)	5.6 ± 1.6	5.3 ± 1.7	0.515	5.5 ± 2.1	4.8 ± 1.8	0.151	0.882
E/Em ratio	12.5 ± 3.6	12.5 ± 3.3	0.872	12.2 ± 5.8	12.3 ± 2.9	0.898	0.875
Speckle tracking echocardiography							
Twist (°)	8.0 ± 3.8	6.8 ± 3.8	0.242	10.1 ± 5.2	8.6 ± 3.5	0.492	0.278
Twist velocity (°/s)	14.7 ± 13.2	13.3 ± 11.2	0.792	17.1 ± 13.2	14.5 ± 14.5	0.740	0.673
Peak untwisting velocity (°/s)	−73.8 ± 30.4	−49.7 ± 23.8	0.006	−64.6 ± 25.5	−68.2 ± 23.5	0.637	0.475
Time to peak untwisting velocity (s)	0.07 ± 0.08	0.09 ± 0.08	0.431	0.10 ± 0.06	0.08 ± 0.07	0.509	0.389
GLS (%)	−19.2 ± 4.7	−19.5 ± 3.9	0.661	−20.6 ± 3.0	−20.6 ± 2.4	0.938	0.480
GCS (%)	−27.2 ± 6.7	−26.5 ± 5.9	0.476	−27.5 ± 6.5	−28.5 ± 4.2	0.657	0.916
Early GLSR	0.90 ± 0.28	0.89 ± 0.19	0.809	0.87 ± 0.21	0.91 ± 0.26	0.460	0.625
Late GLSR	0.76 ± 0.25	0.77 ± 0.24	0.840	0.86 ± 0.19	0.92 ± 0.22	0.429	0.188
Early GCSR	1.91 ± 0.56	1.81 ± 0.50	0.339	1.81 ± 0.75	1.77 ± 0.57	0.883	0.736
Late GCSR	1.26 ± 0.53	1.15 ± 0.33	0.320	1.62 ± 0.34	1.62 ± 0.48	0.990	0.094

Values are mean ± SD

*LA* Left atrial; *LAVI* left atrial volume index; *IVSd* interventricular septum thickness (diastole); *LVPWd* left ventricular posterior wall thickness (diastole); *LVEDD* left ventricular end-diastolic dimension; *LVESD* left ventricular end-systolic dimension; *LVEF* left ventricular ejection fraction; *LVMI* left ventricular mass index; *E* peak early phase filling velocity; *A* peak atrial phase filling velocity; *DET* deceleration time; *Em* peak early wave velocity. Twist is defined as the instantaneous left ventricular peak systolic twist. The peak untwisting velocity is the peak diastolic de-rotation velocity. *GLS* Global longitudinal strain; *GCS* global circumferential strain; *GLSR* global longitudinal strain rate (early and late diastole); *GCSR* global circumferential strain rate (early and late diastole)

^a^Standard error of the difference

### Predictors for response

No association was found between the clinical characteristics, conventional echocardiographic and speckle tracking parameters which could predict response.

## Discussion

The aim of this study was to assess if RDN resulted in functional and structural cardiac changes as assessed using both conventional 2D echocardiography and 2D STE. A secondary objective was to assess any differences in these parameters between responders and non-responders. We observed a significant difference in blood pressure and heart rate at 6 months post procedure. Echocardiographically, at 6 months, a significant difference was noted in LVPWd and A-wave velocity. Additionally, STE demonstrated a significant difference in peak untwisting velocity.

Persistent sympathetic nervous system hyperactivity plays a critical role in hypertension and is associated with significant structural and functional cardiac changes [16]. In this study, we found that RDN significantly reduced office blood pressure by 18.9 ± 26.8/8.5 ± 13.5 mmHg (*p* < 0.001) and heart rate by 4.5 ± 9.9 beats/min (*p* = 0.016). In line with these findings, we observed a significant decrease in LVPWd (0.47 ± 1.0 mm), along with a reduction in LVMI of 3.2 ± 11.6 g/m^2^ in the total cohort. The fact that this reduction did not reach statistical significance could be due to a lack of power.

Looking further into diastolic function, which is strongly related to hypertension and blood pressure control, revealed a pseudonormal diastolic dysfunction, based on normal E/A ratio, but increased left atrial (LA) dimension and E/Em ratio in the overall study population. We observed a significant decrease in the A-wave velocity by 5.3 ± 13.2 cm/s (*p* = 0.044) in the total population. In the responders, the difference in the A-wave velocity even decreased to 8.5 ± 13.8 cm/s (*p* = 0.027). The decrease in A-wave velocity after renal denervation could implicate an improvement in the LV relaxation and a subsequent better diastolic function [17]. Additionally, a pseudonormal diastolic function may reflect a decrease in LV compliance and a moderate increase in LA pressure in our population, with impaired relaxation and prolonged A-wave velocity before treatment. The link between peak untwisting velocity and A-wave velocity may be explained by the rate of uncoiling. In other words, it is likely that less force is needed for the active atrial contraction during late diastolic filling after blood pressure lowering. The relation between LV untwisting and the conventional parameters was also described in previous work, in which a positive correlation between untwisting rate and A-wave velocity was found, while there was no correlation between E-wave velocity and untwisting rate [8]. However, these findings should be interpreted with caution, as no differences were noted in other echocardiographic parameters determining diastolic function, such as the E/Em ratio and LA dimensions.

Hypertensive patients have a greater risk of developing cardiac fibrosis, myocyte hypertrophy and diastolic dysfunction [18]. These changes may influence the LV twist and rotation [19]. LV twist is a wringing motion of the heart as the apex rotates with respect to the base around the LV long axis, which is a key element for regulating LV systolic and diastolic mechanics [20]. LV twist is derived from the dynamic interaction between subendocardial and subepicardial myocardial fibres, the latter defining the direction of the LV twist. It is known that, in myocardial fibrosis and LV hypertrophy, which is related to pressure overload, impairment appears frequently in the subendocardial layer, leading to a dominance of the subepicardial fibres [21]. This might explain the increased LV twist in patients with LV hypertrophy. LV untwisting starts after the peak LV twist. In a healthy population, the peak systolic twist is supposed to store potential energy and is thought to contribute towards diastolic suction and facilitate early LV diastolic filling. Previous work from our group demonstrated that peak untwisting velocity is increased in hypertrophic cardiomyopathy patients with mild diastolic dysfunction, as well as in aortic stenosis patients [8, 22]. Our study is the first to demonstrate a significant decrease in peak untwisting velocity of 14.5 ± 28.9°/s (*p* = 0.025) in patients undergoing RDN. This change was mainly driven by a change in peak untwisting velocity by 24.1 ± 28.7°/s (*p* = 0.006) in the responders. In non-responders, no difference following RDN was observed. The decrease in untwisting velocity after renal denervation could implicate an improvement in the LV relaxation and a subsequent better diastolic function, similar to A-wave velocity. One may hypothesise that, in hypertension patients, like in hypertrophic cardiomyopathy and aortic stenosis patients, increased peak untwisting velocity serves as a compensatory mechanism for abnormal relaxation and prevents the need to increase LA pressure [22, 23]. RDN probably leads to an improvement of these specific changes in LV rotational and de-rotational mechanics, especially in responders. However, no significant change was seen in twist and twist velocity. Finally, we observed a significant decrease in heart rate following RDN in our population. This is in line with several randomised controlled studies which also demonstrated a decrease in heart rate following RDN [24, 25]. Interestingly, a similar decrease in heart rate was observed in the (sham) control arm of both studies, suggesting that RDN by itself has no significant effect on heart rate. Additional exploratory analyses in our study ruled out a correlation between heart rate and untwisting velocity (*R* ^2^ = 0.1%).

Future studies, comparing hypertensive patients with a healthy control group, may be warranted in order to investigate myocardial geometry changes in hypertensive subjects in the context of diastolic LV twisting and untwisting, blood pressure or heart rate independently.

## Limitations

Data are derived from a small patient population and a lack of power might have impacted our findings. Furthermore, accurate assessment of changes in cardiac systolic and diastolic function and volumes with conventional echocardiography produces limited image quality in patients with a body mass index (BMI) above 25 kg/m^2^. In some patients (*n* = 6), automatically detected LV contours had be corrected manually, which might have impacted the reliability of the measurements. Four different renal denervation systems were used. Based on individual previous studies, the blood pressure-lowering effect of these individual devices remains in the same range; however, a differential effect on the echocardiographic parameters measured in our study could not be excluded.

## Conclusion

Renal sympathetic denervation (RDN) reduced blood pressure and heart rate, and significantly improved functional myocardial parameters such as A-wave velocity and peak untwisting velocity in patients with treatment-resistant hypertension, suggesting potential pleiotropic beneficial effects of renal sympathetic denervation on myocardial mechanics. Further dedicated studies are needed to elucidate the potential role of RDN on echocardiographic parameters.
